# Understanding and mathematical modelling of cellular resource allocation in microorganisms: a comparative synthesis

**DOI:** 10.1186/s12859-021-04382-3

**Published:** 2021-09-28

**Authors:** Hong Zeng, Reza Rohani, Wei E. Huang, Aidong Yang

**Affiliations:** 1 Beijing Advanced Innovation Center for Food Nutrition and Human Health, Beijing Technology and Business University, Beijing, 100048 China; 2 Department of Engineering Science, University of Oxford, Parks Road, Oxford, OX1 3PJ UK

**Keywords:** Resource allocation, Proteome allocation, Bacterial growth, Metabolic network, Constraint-based metabolic modelling, Synthetic biology

## Abstract

**Background:**

The rising consensus that the cell can dynamically allocate its resources provides an interesting angle for discovering the governing principles of cell growth and metabolism. Extensive efforts have been made in the past decade to elucidate the relationship between resource allocation and phenotypic patterns of microorganisms. Despite these exciting developments, there is still a lack of explicit comparison between potentially competing propositions and a lack of synthesis of inter-related proposals and findings.

**Results:**

In this work, we have reviewed resource allocation-derived principles, hypotheses and mathematical models to recapitulate important achievements in this area. In particular, the emergence of resource allocation phenomena is deciphered by the putative tug of war between the cellular objectives, demands and the supply capability. Competing hypotheses for explaining the most-studied phenomenon arising from resource allocation, i.e. the overflow metabolism, have been re-examined towards uncovering the potential physiological root cause. The possible link between proteome fractions and the partition of the ribosomal machinery has been analysed through mathematical derivations. Finally, open questions are highlighted and an outlook on the practical applications is provided. It is the authors’ intention that this review contributes to a clearer understanding of the role of resource allocation in resolving bacterial growth strategies, one of the central questions in microbiology.

**Conclusions:**

We have shown the importance of resource allocation in understanding various aspects of cellular systems. Several important questions such as the physiological root cause of overflow metabolism and the correct interpretation of ‘protein costs’ are shown to remain open. As the understanding of the mechanisms and utility of resource application in cellular systems further develops, we anticipate that mathematical modelling tools incorporating resource allocation will facilitate the circuit-host design in synthetic biology.

## Background

Following the initial suggestion in 2009 by Molenaar et al*.* [1] that cellular growth strategies are dependent on not only metabolism but also the synthesis cost of proteins, extensive efforts have been made to investigate the biophysical importance of the allocation of macromolecular resources in supporting cell growth. Significant progresses were obtained in the past decade. Quantitative analysis of growth-dependent proteomic datasets elucidates that resource allocation plays a central role in dictating metabolism and gene expression for maximizing the rates of steady-state growth [2–5]. Resource allocation has also been shown to govern transitional growth kinetics upon the nutrient shift in a global manner [6].

Several review articles have been produced in the past few years to consolidate the knowledge base of resource allocation. These include an insightful revisit of the history of the idea of resource allocation in living organisms and the development of resource balance analysis (RBA) model [7]. Another work demonstrates the power of multiscale metabolic models and omics datasets in elucidating resource allocation principles [8]. Complementary to [8] which focuses on fine-grained models that integrate metabolic networks with gene expression, a separate review of phenomenological, i.e. coarse-grained resource allocation models presents the value in making quantitative predictions of microbial phenotypes with only a few adjustable parameters [9]. Furthermore, a recent review summarises the mathematical structures of models that can predict the overflow metabolism, unifies all models into one standard form and concludes that two growth-limiting constraints are essential for predicting the gradual switch from a high-yield to a low-yield pathway [10].

Complementary to the existing reviews, this paper adds to the synthesis of the development of this area by (1) offering a generalised conception of cellular resource allocation, (2) presenting the converging understanding of the role of resource application in achieving cellular objectives, (3) classifying and contrasting proposed root causes of proteomic resource allocation, (4) identifying the commonalities and contrasts of both predictive and descriptive mathematical models that incorporate resource allocation, (5) exploring the implications of resource allocation research for synthetic biology, and (6) finally highlighting open questions in both understanding and mathematical modelling. We posit that resource allocation can help answer central bioengineering questions such as how microbes determine growth strategies in a changing environment and what overarching governing principles can guide the design of microbial factories. As such, an application-oriented outlook is also given. Overall, we hope that this concise review will add clarities to the understanding of what has been achieved in this area and hence facilitate its future development.

## Types of resources for living microbial cells and the general concept of resource allocation

### External and internal resources

Microorganisms are unicellular organisms that require a range of resources to maintain their viability and to grow and self-replicate. These resources can be sorted to external and internal resources. External resources are environmental provisions that can be utilised by the cell, e.g. chemical substrates (organic compounds, carbon dioxide), nutrients and light. Internal resources comprise those that a cell ‘owns’, such as genetic information, cellular machinery (e.g. ribosomes, RNA polymerases (RNAPs), enzymes and other RNA- or protein-based molecular catalysis) and spatial resources such as membranes and intracellular space. Generally, resource allocation may refer to the cell’s dynamic allocation of any types of internal resources for certain objectives. Possibly because ~ 60% of the dry cell weight are proteins [11–13] and ~ 85% of the extracellular resources are used for protein productions [14], the allocation of proteomic resources has become the most acknowledged and best-studied among all the resource types.

While in theory external resources can be infinitely supplied, internal resources generally have physical limitations. Fundamentally this limitations arise from the fact that cells are self-replicating systems, at least during steady-state growth, so only certain combinations of parameter values are permissible [15, 16]. For example, the limited translational speed (~ 40 amino acids per second per ribosome [8]) intuitively calls for an adequate allocation strategy (of finite ribosomal machinery) for fast growth (or upon nutrient shift) where numerous additional copies of proteins are required. Besides, there are often close links between internal and external resources. Importing extracellular substrates, e.g. glucose, into the cytoplasm requires various transporters (internal resources) [17]. Free energy (internal resource) needed to fuel biological processes is generally extracted from organic compounds or light (external resources) via respiration (or photosynthesis) and is stored and transferred mostly via ATP molecules. Furthermore, cellular membranes isolate the cell from the outer environment, allowing a relatively stable and mild internal space to house fragile and delicate biological apparatus. Surface membranes also accommodate a variety of proteins that control the mass and information transfers between internal and external environments.

### A putative decision-making process for resource allocation

**Fig. 1:**
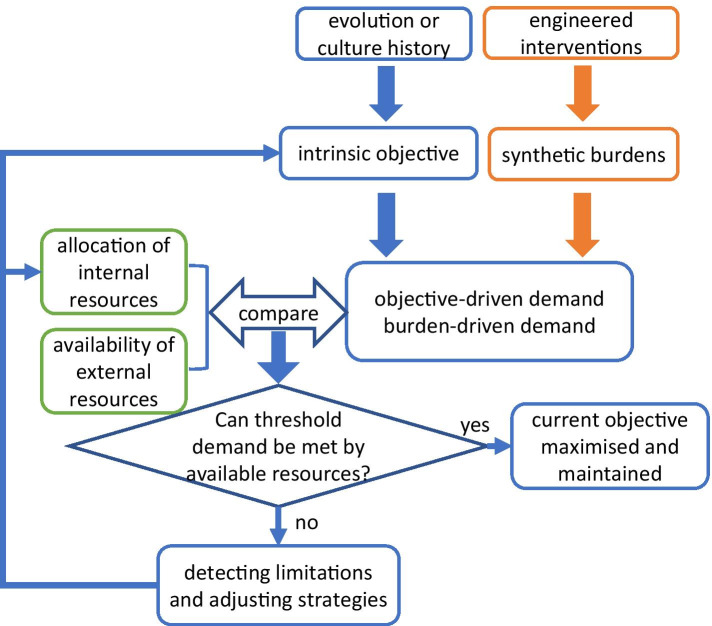
Illustration of a putative decision-making process underlying the observed dynamic allocation of cellular internal resources. The evolution or culture history normally confers the cell an intrinsic objective, e.g. maximisation of growth, which forms objective-driven demands. For engineered organisms, additional demands can originate from synthetic burdens. If the demands can be met by available external and internal resources, the current objective will be achieved. Otherwise, the cell can detect limitations and devise response strategies, e.g. modulating its internal resources, or under certain circumstances, reconfigure its objective

We postulate that many of the observed dynamic allocations of cellular (internal) resources in microorganisms are attributable to the balance between the cellular demand and the supply capability (Fig. 1). At any point in time, a microbial cell normally pursues an intrinsic objective, e.g. maximisation of growth, due to evolution or culture history. However, the level at which the cell actually achieves this objective depends on the extent to which the corresponding demands for materials and energy are met. Within an engineered organism, additional demands can originate from burdens of synthetic gene-circuits. For instance, the expression of recombinant genes requires additional building molecules, energy and expression machinery [18]; sub-lethal antibiotic dosage provokes the need for more ribosomes to maintain growth [2]. With certain (possibly changing) external and internal environments, the cell can be viewed as constantly facing a question as to whether the external and internal resources available to the cell can fulfil the requirement to achieve its chosen objective at a minimal threshold level. If the demands of this threshold are sufficiently met by the available resources, the current objective is pursued. Otherwise, a ‘limited’ or ‘stressed’ state will materialise (note that the term ‘stressed state’ here includes stresses that are driven by the shortage of internal and/or external resources, which is more general than the environmental stress often used in biology), which can be detected by the cell and lead to certain responses through multiple regulatory mechanisms [14, 19]. Under certain circumstances, the cell is able to respond to the stress by manipulating its physiology to bypass the limitation without interfering its intrinsic objective. However, if the discrepancy between demand and supply is irreconcilable, the cell may reconfigure its objective, e.g. gradually changing from growth to survival to compromise. The internal resources will be adjusted accordingly to fit to the changed objective.

It has previously been noted that growth rate-dependent regulation is not always dominant [8]. The above putative “active decision-making process” intends to explain the occurrence of resource allocation under both nutrient-scarce (where growth is often not the first priority) and nutrient-rich growth conditions (where the cell grows at fast rates). Reflecting on previous discussions in this broad area (e.g. in [14] and [8]), we posit that cellular objectives, burdens and limitations (or stresses) are closely entangled and together contribute to the tug of war between supply and demand; it seems neither logical nor feasible to discuss one concept independently without considering the others. Applying orthogonal perturbations [9] (and adaptive laboratory evolution (ALE) if stress-driven mutations are of interests [20]) would help capture the transition between distinct cellular objectives to uncover the comprehensive decision-making atlas underlying the changing phenotypes across different growth conditions.

## Current understanding of resource allocation in microorganisms

### Resource allocation decisions facilitate cellular objectives

In the past decade, our knowledge in resource allocation thrives based on the study of bacteria’s ability to actively modulate their proteome compositions to maximise the steady-state growth rate [2–5]. A typical example of growth rate-driven reallocation of proteome is the preference of carbon-spilling fermentation pathway in fast-growing *Escherichia coli*. As growth rate increases, *E. coli* changes its metabolic strategy from carbon-efficient respiration to proteomic-efficient aerobic fermentation (for energy biogenesis) so that more proteomic resources can used for biomass synthesis to support rapid growth [4]. However, as pointed out in [8], growth rate-dependent regulations are not always dominant. The proteome of *E. coli* grown on pyruvate, glycerol and galactose is not optimized for fastest growth [21]. Besides, a proteomic study of ‘persisters’ (i.e. bacteria with transient antibiotic tolerance) [22] reveals that resource allocation plays a key role in coordinating metabolism towards maximizing energy yield, instead of biomass production. The specific proteome adjustment observed in persister cells, which is comparable with that in starved and stressed cells, was shown to be driven by increased ppGpp levels (a result of general stress or stringent response) rather than a mere consequence of reduced growth rate [22]. Furthermore, it has proven that strong environmental perturbations can alter the protein composition through directly or indirectly interfering protein synthesis processes [23].

**Fig. 2:**
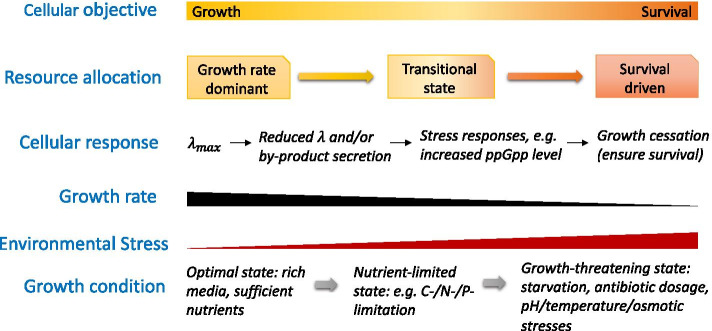
The relationship between cellular objective and resource allocation with changing growth conditions. The environmental stress increases as growth conditions vary from optimal (sufficient nutrients to support the maximal growth) to nutrient-limiting, e.g. in the case of carbon, nitrogen or phosphorus limitation and finally to growth-threatening (strong perturbations on bacterial homeostasis). During this process, the growth rate reduces from the maximal value ($\lambda_{max}$) to almost zero (i.e. the cell stops growing). The signal molecule ppGpp accumulates as a result of the general stress or stringent response [22]. The cellular objective shifts from maximizing growth to ensuring bacterial survival. Subsequently, resource allocation becomes less coupled with the growth rate and eventually functions for ensuring survival

Since mounting evidence suggests that variations in the proteome composition (a typical example of resource allocation as stated above) can result from the cell fighting against vicious threats in addition to coping with rapid growth, it is reasonable to depict the resource allocation phenomena along with the cellular objective given certain growth conditions. Growth conditions can change from an optimal state (where the external nutrients efficiently support maximal growth), through a nutrient-limited state (where a cell can still grow but at a reduced rate or needs to adjust its physiology, e.g. secretion of a by-product, to maintain the same growth rate) and finally to a growth-threatening state (e.g. under osmotic, pH and temperature stresses where the cell could only maintain slow growth or even completely stop growing [24]) (Fig. 2). The cellular objective can correspondingly shift from growth to survival. Accordingly, it can be postulated that resource allocation emerges to increase cellular fitness to the changing growth environments. As the growth condition becomes increasingly harsh, resource allocation will become less coupled with the growth rate and eventually function for ensuring survival.

### Pre-allocation and growth optimality

Apart from dynamically facilitating cell growth and survival, resource allocation can take the form of pre-allocation which reflects its evolutionary importance. For instance, *E. coli* is able to pre-allocate its proteome. It has been shown that a considerable amount of proteins in *E. coli* is expressed with no immediate benefit given specific growth conditions [25]. Besides, many catabolic genes for substrates that are not presented in the medium were found to be upregulated with decreasing carbon quality [3, 5]. In addition to protein pre-allocation, carbon-limited cells have been shown to have a higher fraction of inactive ribosomes (i.e. free ribosomes not bond to mRNA) than phosphorus-limited cells, which allows rapid growth acceleration upon nutrient upshift [26]. Pre-allocation of ribosomal capacity was also observed in cells undergone famine-to-feast cycles and is considered beneficial to the overall gain of biomass [27]. Furthermore, a recent modelling work quantitatively shows that spare ribosomal capacity prevents metabolic overshoots and permits rapid response to nutritional upshifts [28].

While it is generally considered that constitutive pre-allocation of cellular resources (proteins and/or ribosomes) can provide preparatory advantages to hedge against sudden environmental changes [29], such strategic decision also imposes substantial burdens that prevent the cell to grow faster. This may reflect an evolutionary choice of maintaining higher robustness at the cost of slower growth rates. Despite the suggestion that the inability to grow at the fastest rate could be explained by limited regulatory capabilities [9], it is also possible that a seemingly ‘sub-optimal’ growth strategy that enables better response upon nutrient shifts is in fact the ‘optimal’ strategy selected by evolution. From the evolution point of view, it might be inappropriate to evaluate the optimality of a growth or resource allocation strategy by growth rate only. Instead, resource allocation may play a central role on multiple fronts including improving cell growth, ensuring survival and enabling high adaptability and ultimately confer the cell greater evolutionary advantages to compete against others in fluctuating environments.

### RNA and space allocation

The notion of resource allocation introduced above can in principle manifest with other internal resources, such as RNA and subcellular space in addition to proteins. We did not find existing evidence for intrinsic RNA allocation independent from proteome allocation or ribosome allocation (possibly because ~ 85% of the RNA is ribosomal RNA [30, 31]). Nevertheless, artificial controls of mRNA populations [32] and the activity of RNAP [33] to direct the cellular resources to synthetic circuits have been achieved in synthetic biology. On the utilisation of intracellular space, the experimental observations of maximal cell buoyant density [34] and limited membrane protein density [35] imply that the space allocation can happen either over the whole cell or within certain subcellular compartments. In light of natural strategies for the spatial organization of metabolism (e.g. organelles in eukaryotic cells or bacterial microcompartments (BMCs) in prokaryotic cells) [36], there could be more sophisticated strategies for dynamic spatial organisation yet to be discovered.

## Growth rate-driven proteome allocation

### Using proteome allocation to explain important biological phenomena

**Fig. 3:**
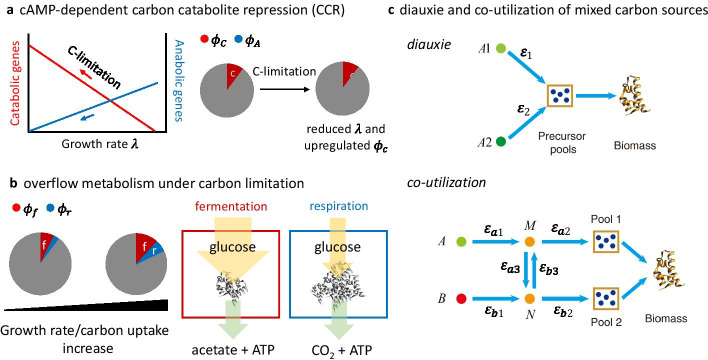
Proteome allocation can explain important biological phenomena. **a** Illustration of the physiological function of cAMP-dependent carbon catabolite repression (CCR) in allocating proteomic resources to meet growth demand adapted from ref. [3]. Under carbon limitation, catabolic genes (namely mass fraction of catabolic proteins $\phi_{C}$) are upregulated while anabolic genes (indicated by the mass fraction of anabolic proteins $\phi_{A}$) are downregulated with decreased growth rates. **b** Illustration of the overflow metabolism in fast-growing *E. coli* adapted from ref. [4]. The fraction of total proteome allocated to fermentation ($\phi_{f}$) and respiration ($\phi_{r}$) is different between slow growth (low carbon uptake) and fast growth (high carbon uptake). The key driver of such modulation of proteome resources lies in the much lower protein investment per ATP flux (yellow arrow) of the fermentation pathway compared with respiration. **c** Illustration of the coarse-grained model of diauxie and co-utilization of carbon sources adapted from ref. [37]. For diauxie, two group A sources (*A1* and *A2*) can both supply precursor pools for biomass production but with different pathway efficiencies ($\varepsilon_{1}$ and $\varepsilon_{2}$). The one with higher efficiency is preferred for maximal growth. If two precursor pools supply the biomass synthesis, each pool derives from an intermediate node *M* or *N*. Either intermediate node can draw flux from either of the two sources *A* and *B*. Co-utilization occurs under conditions where the efficiency for biomass production is highest when directly drawing carbon flux from source *A* to precursor Pool 1 and from source *B* to precursor Pool 2, i.e. the optimal overall efficiency would be $\varepsilon_{a1} + \varepsilon_{a2} + \varepsilon_{b1} + \varepsilon_{b2}$

Being the most acknowledged and best-studied type of resource allocation, growth rate-dependent proteome allocation provides insights for many well-known biological phenomena, e.g. the cyclic adenosine monophosphate (cAMP)-dependent carbon catabolite repression (CCR) [3], the overflow metabolism in fast-growing *E. coli* [4] and the change between diauxie and co-utilization of mixed carbon sources [37]. For CCR, it has been shown that [3] the physiological function of cAMP-mediated CCR is to ensure proteomic resources are invested as needed for bacterial growth under diverse nutrient conditions, e.g. more resources are directed to biosynthetic processes as growth rate increases (Fig. 3a). For overflow metabolism, Basan et al*.* [4] proposed and validated that proteome allocation plays a critical role in regulating the proteomic resources invested between different energy pathways. They quantified that the protein cost per ATP produced by the fermentation pathway is about 67% of that by respiration, which constitutes the key driver of the activation of the fermentation pathway at rapid growth (Fig. 3b). More recently, a coarse-grained model of optimal allocation of protein resources quantitatively explained why and how the cell chooses between diauxie- and co-utilization of substrates under mixed carbon sources [37]. Carbon sources were categorized into those introduced at the upper part of glycolysis (Group A sources) and those entering at other nodes of the metabolic network (Group B sources). Prioritised carbon utilization occurs among Group A sources, and usually the one that supports higher growth rate (associated with higher substrate quality and higher pathway efficiency) is preferred. The preference of carbon sources is usually regulated by catabolic repression which repressors inhibit the gene expressions for catabolism of unfavourite carbon sources [38]. Sometimes, when Group A and Group B sources are both present in the media, co-utilisation would arise if it is more economical for some precursor pools to take a shortcut of drawing carbon flux from Group B sources while for other precursor pools taking up Group A sources is more efficient (Fig. 3c). Furthermore, the remarkable achievement of using resource reallocation to explain the overflow metabolism in *E. coli* sheds light on the mechanisms for other widely recognised overflow phenomena, e.g. the production of ethanol in *Saccharomyces cerevisiae*, i.e. the Crabtree effect [39–41] and the production of lactate in cancer cells, i.e. the Warburg effect [42–44].

### Investigating the potential physiological root cause of the overflow metabolism

Although various studies [1, 4, 14, 41, 45–47] have proven that proteome allocation plays a crucial role in regulating the overflow metabolism in *E. coli*, they are not without controversies. In particular, the macromolecular crowding (also known as ‘molecular crowding’) hypothesis [45, 46] and the constrained proteome allocation hypothesis [4] have been considered hard to reconcile with each other [9]. In this section, we discuss different hypotheses for explaining the overflow metabolism towards deciphering the potential physiological root cause.

Molecular crowding hypothesis is based on the notion that a cell has an upper limit or optimal macromolecular density [48]. It proposed that the hard bound on the intracellular macromolecule concentration (or equivalently the finite cell volume or the solvent capacity constraint) triggers the metabolic shift from full respiration to the overflow metabolism [45, 46]. This proposition is supported by the observed change in the cell buoyant density of *E. coli* MG1655, which gradually increases when growth rate increases from 0.1 to 0.4 h^−1^ and stays roughly constant at higher growth rates [34]. Therefore, the physiological root cause of the overflow metabolism in molecular crowding is hypothesised to be the finite cell buoyant density, which in this work is further classified as space limitation (Table 1).

**Table 1:** Comparison between competing proteome allocation-based hypotheses and models for explaining the overflow metabolism

	Molecular crowding	Constrained proteome allocation	Membrane occupancy	RBA and ME
Physiological root cause	Finite cell volume	Un-specified	Finite membrane area	Limited translational efficiency and/or limited catalytic rate of enzymes
Global or local regulation	Global	Global	Local	Global
Types of limitation	Space limitation	Un-specified	Space limitation	Machinery limitation
References	[45, 46]	[4]	[35, 47]	[52, 54]

On the other hand, the researchers developing the constrained proteome allocation hypothesis observed significant changes in the proteome composition, in particular for energy biogenesis, upon the metabolic shift from normal growth to the overflow metabolism in *E. coli* NCM3722 [4]. As mentioned above they determined that the protein investment per ATP flux of fermentation is about twice as efficient as that of respiration. This leads to their critical argument that the overflow metabolism results from the cell’s preference of more proteomic efficient pathways at rapid growth. However, although the work showed an extrapolated upper bound (from the proteomic data) of the proteome fraction available for energy biogenesis, an indication is lacking as to what physiological constraint leads to this phenomenological limitation (more discussion is provided in the section below).

Reflecting on the molecular crowding hypothesis, Basan et al*.* [4] argued that the macromolecular density (or the cell volume) constraint is not a valid constraint [4, 9] as (1) the cell volume varies widely between growth conditions with similar densities [49] and (2) they did not observe variations in cell density (within a wide range of growth rates) in their own measurements [50]. In a subsequent discussion, Vazquez and Oltvai from the molecular crowding ‘camp’ suggested that the prediction of the overflow metabolism by the constrained proteome allocation model in [4] is achieved through ‘implicit assumptions that expand beyond the hypothesis of proteome allocation alone’ [48]. More specifically, they showed mathematically that in addition to the differential proteome efficiencies between fermentation and respiration, the prediction of the acetate production flux also requires (1) “a non-zero density of non-metabolic macromolecules”, and (2) “an upper bound in the cell macromolecular density”. They further stated that molecular crowding “explains” the latter point and hence “is a key factor in explaining overflow metabolism”.

Part of the above debate is concerned with cell densities at different growth rates. It should be pointed out that the reported range of growth rates with constant cell density for *E. coli* NCM3722 (in the constrained proteome allocation hypothesis) is 0.3–2.0 h^−1^ [50], and the overflow metabolism of this strain occurs at growth rates above ~ 0.8 h^−1^ [4]. On the other hand, the reported growth rate range of *E. coli* MG1655 (in the molecular crowding hypothesis) is 0.1–0.7 h^−1^ with the acetate overflow occurring at growth rates above 0.4 h^−1^ [45]. For MG1655, the cell buoyant density increases with the growth rate within the range of 0.1 to 0.4 h^−1^ and plateaus at growth rates above 0.4 h^−1^ [34]. One can see several key differences between these two cases in terms of the strain, the range of growth rate, and the growth rate at the onset of overflow; these differences call for cautions when comparing alternative propositions.

Other mathematical models incorporating proteome allocation-derived constraints imply additional root cause of the overflow metabolism. FBA^ME^ (membrane economics) [47] suggests that the simultaneous use of fermentation and respiration at high growth rates is an outcome of finite cytoplasmic surface area (available for respiratory membrane proteins), which is classified here as (local) space limitation (Table 1). The membrane economics hypothesis is reinforced by a recently proposed membrane real estate hypothesis [35], with the presentation of experimental evidence of the decrease in surface-to-volume ratio and limitation on membrane-protein packing capacity at increased growth rates. However, Basan questioned the membrane economics hypothesis in its inability to explain the overflow metabolism emerging in slow-growing cells expressing a large amount of useless proteins, where the membrane capacity should be sufficient [9]. Additionally, Resource Balance Analysis (RBA) [14, 51, 52] and Metabolic and macromolecular expression (ME) [53, 54] models indicate that the change in the macromolecular composition (primarily proteins) and the occurrence of the overflow metabolism at increased growth rates are derived from limited synthesis capacity of macromolecules (e.g. limited translational rate) and limited efficiencies of molecular catalysis (e.g. limited enzymatic catalytic rates), which is classified here as machinery limitation (Table 1).

The proposals described above suggest that the root cause (i.e. fundamental physiological limitation) of the overflow metabolism and the accompanied proteome re-allocation is not a concluded matter. At least two competing explanations, i.e. space limitations (represented by molecular crowding [45, 46] and membrane occupancy [35, 47] hypotheses) and machinery limitations (represented by RBA [14, 52], ME [53, 54] and possibly implicitly reflected by constrained proteome allocation hypotheses [4]) have been supported by experiments and/or mathematical models at least to a certain degree (Table 1). Besides, it is worth noting that molecular crowding, constrained proteome allocation hypotheses, RBA and ME correspond to global regulations of the proteome whereas the membrane occupancy-based hypotheses can only explain local proteome adjustments.

### The potential link between the proteome fractions and the partition of ribosomal machinery

Interested in the possible physiological root cause behind the constrained proteome allocation hypothesis, we conducted the following derivations to understand how the proteome composition can be linked to the ribosomal machinery. The time-dependent change of the concentration of protein $i$ ($P_{i}$) can be modelled as protein generation (via ribosome translation) minus dilution and degradation rate.

1 $$\frac{{dP_{i} }}{dt} = r_{i} - \lambda P_{i} - D_{i} P_{i}$$

$r_{i}$ is the generation rate of protein $i$, which is proportional to the ribosome abundance ($R$), i.e. $r_{i} \propto R$ [55]. $\lambda P_{i}$ is the protein dilution term with $\lambda$ being the specific growth rate.$D_{i}$ is the degradation rate of protein $i$ [56, 57]. We define the partition (allocation) of ribosomal machinery $\theta_{i}$ as the production rate of protein $i$ divided by the overall production rate of proteins.

2 $$\theta_{i} = \frac{{r_{i} }}{{\mathop \sum \nolimits_{i} r_{i} }}$$

At steady-state exponential growth, $dP_{i} /dt = 0$ and $\lambda$ is constant, Eq. 1 implies

3 $$r_{i} { } = \left( {\lambda + D_{i} } \right)P_{i}$$

Substituting Eq. 3 into Eq. 2 gives

4 $$\theta_{i} = \frac{{\left( {\lambda + D_{i} } \right)P_{i} }}{{\mathop \sum \nolimits_{i} \left( {\lambda + D_{i} } \right)P_{i} }}$$

The average $D_{i}$ for non-ribosomal proteins (NRPs) has been reported to be ~ 0.1 per total NRPs per hour [55]. In growing *E. coli*, about 40% mass of total proteins is stable while the half-life of most of the remaining proteins is between 15 and 30 h, corresponding to degradation rate of 0.05–0.02 h^−1^ [58]. During steady-state exponential growth, especially for acetate-producing *E. coli* where the specific growth rate can readily reach beyond 0.8 h^−1^ [4], therefore it is reasonable to assume $\lambda \gg D_{i}$. The protein degradation term thus can be considered negligible compared with the growth dilution effect. Equation 4 can be simplified as

5 $$\theta_{i} = \frac{{\lambda P_{i} }}{{\mathop \sum \nolimits_{i} \lambda P_{i} }} = \frac{{P_{i} }}{{\mathop \sum \nolimits_{i} P_{i} }} = \phi_{i}$$

where $\phi_{i}$ denotes the proteomic fraction of sector $i$ [4]. It is worth noting that Eq. 5 is generally valid for fast-growing cells. Cells under strong burdens or environmental perturbations may not strive for maximal growth. Equation 5 should not be applied to these scenarios.

A direct interpretation of Eq. 5 is that the division of ribosomal machinery is approximately equal to the proteome composition if the cell is growing in the exponential phase (i.e. steady-state rapid growth). More importantly, if the proteome fractions can be seen as a direct proxy of the partition of ribosomal machinery, the hard proteomic constraint proposed by Basan et al.[4] can result from translational limitations, not necessarily belonging to a special case of the molecular crowding hypothesis. it should be noted that $D_{i}$ has been reported to vary considerably between different proteins in a cell, e.g. in *E. coli* [58] and in *Lactococcus lactis* [59]. At slow growth where dilution becomes comparable with protein degradation, the discrepancy in $D_{i}$ of individual proteins could become impactful which would in turn affect the validity of the approximate equivalence between proteome composition and ribosome occupancy. Nevertheless, this analysis shows that the proteome composition and observed proteome allocation constraints [4] may closely link to the allocation of the ribosome machinery, and this dependency is particularly strong in steady-state rapid growth. It is worth noting that a formulation similar to Eq. 5 has been shown in a recent study [60], which proposes that at steady state the relative strength of resources recruitment of a given protein equals its relative mass in the cell; this relevant work also links the maximum growth rate at steady state with the fraction of ribosomes being used to build new ribosomes relative to the total number of ribosomes, which resembles the fraction $\theta_{i}$ shown in Eq. 5. On the other hand, the above mathematical derivation shows the approximate equality between $\theta_{i}$ and $\phi_{i}$, but it does not provide any evidence for the root source of the ‘limitation’ observed over $\phi_{i}$, which can potentially derive from either (1) the shortage of ribosomal machinery $\theta_{i}$, which triggers the cell’s regulation of protein synthesis, or (2) the shortage of space, which triggers the cell’s regulation of protein synthesis; as part of the response, $\theta_{i}$ will be adjusted, leading to the change in $\phi_{i}$. Therefore, further elucidation remains necessary to increase our understanding on the potential causal links.

## Network-based mathematical models of cell growth and metabolism incorporating resource allocation

### Predictive and descriptive models

**Fig. 4:**
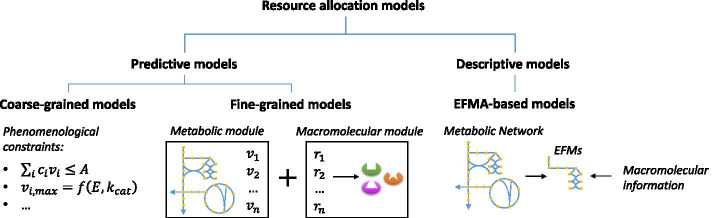
Network-based metabolic models incorporating resource allocation (RA). RA models were classified into predictive and descriptive models. Predictive RA models include coarse-grained models that incorporating phenomenological constraints of macromolecular expressions and fine-grained models that integrate metabolic models with detailed matrices of macromolecular expression processes. Descriptive models are EFMA-based RA models that investigate resource allocation phenomena through converting the metabolic network into EFMs and combining EFMs with macromolecular expression information

We classify network-based metabolic models incorporating resource allocation (RA) into two major categories, i.e. predictive and descriptive RA models (Fig. 4). Predictive RA models are primarily Flux Balance Analysis (FBA [61])-based models for recapitulating growth phenotypes, which can be further classified into coarse-grained and fine-grained RA models, as distinguished in previous reviews [8, 9].

Coarse-grained RA models incorporate resource allocation as phenomenological constraints to constrain the solution space of metabolic fluxes. The constraint can be in the form of imposing an upper bound ($A$) to the sum of the product of metabolic flux ($v_{i}$) and its ‘cost’ ($c_{i}$), i.e. $\mathop \sum \limits_{i} c_{i} v_{i} \le A$ [41, 45–47, 62–64], where $i$ denotes a flux to be included in the constraint. Alternatively, the constraint can be expressed as the maximum reaction rate ($v_{max}$) being a function of the enzyme abundance ($E$, limited by an upper bound based on absolute proteomics) and the turnover rate ($k_{cat}$, queried from BRENDA [65]), i.e. $v_{max} = f\left( {E,k_{cat} } \right)$ in GECKO [66]. The simple form of phenomenological RA constraints allows coarse-grained RA models easy to construct. In contrast, fine-grained RA models integrate metabolic models with a macromolecular expression module, where the transcriptional and translational processes are described in the mechanistically detailed manner. Examples of fine-grained RA models are RBA [14, 51, 52] and ME [53, 54], as well as the more sophisticated whole-cell model of *Mycoplasma genitalium* [67]. The enhanced mechanistic nature makes fine-grained models useful in fundamental studies and biological discovery, e.g. identifying the ‘core proteome’ that must be expressed to sustain cell growth [68] and to compute the metabolic cost of the production of virulence factors for plant pathogens [69]. It is worth noting that, although fine-grained models are more informative, their construction tends to be more demanding which could limit their applications to complex systems. In contrast, coarse-grained models offer a compromise between the level of details and the range of applicability. Therefore, the choice between these two types of models may depend on the application context. In addition to static coarse-grained and fine-grained RA models, efforts have been made to fuse enzyme production costs into dynamic frameworks. For example, deFBA [70] offers a dynamic optimization approach that explicitly includes detailed description of biomass composition and accounts for related enzyme capacity constraints. It was developed to solve dynamic flux optimization problems for metabolic networks coupled with gene expression. Extended from classical dynamic FBA (dFBA [71]) and static RA models, the deFBA method can predict the dynamics of both metabolic fluxes and biomass composition during metabolic adaptations.

Distinguished from the above predictive models, resource allocation have been incorporated with another stoichiometric modelling approach of metabolic networks (additional to FBA), i.e. Elementary Flux Mode Analysis (EFMA [72]). EFMA extracts the most ‘essential’ components of a metabolic network, termed the Elementary Flux Mode (EFM), which comprises a minimal set of enzymes that could operate a non-decomposable set of fluxes at steady state [72]. By overlaying biomass-producing EFMs with enzyme information, e.g. molecular weight, amino acid sequence, EFMA-based RA models were able to associate the occurrence of the overflow metabolism with the cell’s preference of low elemental requirements to construct a functional metabolic pathway [73] and growth rate-yield trade-offs [74]. Furthermore, by deciphering the observed optimal metabolic flux distributions into biomass-producing EFMs combined with resource allocation, it has been mathematically proved that the number of active metabolic pathways (i.e. EFMs) is at most equal to the number of biophysical constraints [75]. For models intended to describe normal and overflow growth states of a cell (corresponding to two active EFMs), at least two biophysical constraints are required. In general, EFMA-based RA models [73–75] do not focus on predicting growth phenotypes, therefore is considered here as descriptive. They are particularly useful for providing a posteriori explanations of resource allocation associated phenotypic patterns, showing how (mathematically) the observed phenomena are consistent with the topology of the metabolic network. Besides, similar to fine-grained models, it could be challenging to construct EFMA-based models for complex systems.

### Key to the predictive power of fine-grained models: adopting variable catalytic rates

In addition to predicting the maximum growth rate and metabolic fluxes as achieved in coarse-grained RA models, fine-grained RA models like RBA [14, 51] and ME [54, 76] are able to predict the abundance of metabolic enzymes, ribosomes and other RNA- or protein-based macromolecules. The improved predictive power is achieved not only through the inclusion of macromolecular expression module, but also results from more delicate treatments of the variable efficiency of molecular machinery.

In RBA, the prediction of cellular configurations in a specific growth condition (e.g. condition X) is achieved by a two-step procedure [52]. The first step is the calibration of the apparent catalytic rate $k_{Ei}$ using flux data (i.e. growth rate, uptake and exertion fluxes) and proteomic data measured from cells grown in condition X. The second step is the growth simulation with the goal of growth rate maximization. To run RBA growth simulations, $k_{Ei}$ are set to the calibrated values (obtained in step one) and the extracellular nutrient concentrations are set corresponding to condition X. If the large datasets (flux and proteomic data) required for $k_{Ei}$ identification (in step one) of another growth condition of interest, e.g. condition Y, is not available, the RBA growth simulation for condition Y is enabled through linear regression and projection of $k_{Ei}$. To do this, several rounds of $k_{Ei}$ calibration (step one mentioned above) needed to be run using available fluxes and proteomic datasets obtained from other growth conditions (with different measured growth rates). The resulting multiple sets of calibrated $k_{Ei}$ are then related to the measured growth rates via a linear function, i.e. $k_{Ei} = a_{i} + \lambda b_{i}$, where $a_{i}$ and $b_{i}$ are linear coefficients. With this estimated linear correlation, RBA growth simulations for cells grown in condition Y can be performed by (a) setting $k_{Ei}$ to the value predicted by the linear equation (noted that $\lambda$ of condition Y should have been measured) and (b) setting the extracellular nutrient concentrations according to condition Y.

In ME [54], two different types of growth simulation are considered: strictly nutrient-limited (SNL) simulation and proteome-limited simulation. In SNL growth simulations, metabolic enzymes are assumed to be operating below their maximal capacity, i.e. $k_{eff} < k_{cat}$, where $k_{eff}$ is the effective catalytic rate (equivalent to the apparent catalytic rate $k_{Ei}$ used in RBA); $k_{cat}$ is the maximal catalytic rate, which is a genuine constant and is set to be proportional to the enzyme’s solvent accessible surface area (SASA). In SNL growth simulations, $k_{eff}$ is a free variable and is predicted together with the maximum growth rate, metabolic fluxes and macromolecular abundances. In proteome-limited growth simulations, metabolic enzymes are assumed to be operating at their maximal rate, i.e. $k_{eff} = k_{cat}$. For both SNL and proteome-limited simulations, the growth conditions are set by specifying the maximum glucose (or another growth-controlling nutrient) uptake rate.

The above description shows that (1) both RBA and ME treat the apparent catalytic rate of metabolic enzymes (i.e. $k_{Ei}$ or $k_{eff}$) as growth rate- or condition-dependent variable, although the concrete modelling treatments are different; (2) the variable catalytic rate plays an important role in predicting the macromolecular states. The validity of this treatment is supported by the qualitative consistency between the experimental data-derived linear correlation $k_{Ei} = a_{i} + \lambda b_{i}$ [52] and the predicted and measured linear $\frac{{k_{eff} }}{{k_{cat} }}vs.\lambda$ profiles of phosphotransferase system (PTS) activity [54]. Besides, this idea might also be useful in improving the model prediction in coarse-grained RA models like CAFBA [62], where the enzyme turnover rate ($k_{cat}$) was assumed constant.

## Other considerations

### Cost, benefit and trade-offs

Resource allocation models discussed above generally associate the physiological burden of proteins (often termed as the metabolic cost, protein cost or enzyme cost) with the protein synthesis process. However, the study of the *lac* operon model system [77] reveals that the *activity* of *lac* permease LacY, not the production or misfolding of the primary protein LacZ, accounts for the major physiological burden (quantified as relative reduction in growth rate due to operon expression) to the cell. The counter-intuitive result found in *lac* operon offers a reminder that future effort on resource allocation, in particular the modelling of ‘protein costs’, needs to take more caution to avoid ambiguous or even misleading interpretation of its biological basis.

In parallel to the cost, the benefit for protein expressions has been defined as increased growth rate [77], increased ATP production rate per protein [4], increased energy efficiency (growth rate divided by ATP production rate) [55], increased product yield [64] and increased adaptability to the changing environments [25, 27–29]. Examining different sets of cost and benefit can lead to the study of different trade-offs, such as those between pathway protein cost and yield [73, 78], growth rate and yield [74, 79] and unused enzymatic and ribosomal capacity and additional storage [80]. These trade-offs may affect growth rates through distinct mechanisms and thus deserve separate attentions.

### Bacterial growth in rich media

Most of the resource allocation studies focus on changes in the phenotypic patterns of cells grown on minimal media. Recent progress in the batch growth of *E. coli* in undefined rich medium reveals the critical role of amino acid catabolism in regulating the central carbon metabolism (e.g. inhibiting glucose uptake and increasing acetate overflow) for faster growth [81]. The reported intricate trade-off between decomposing expensive resources (e.g. methionine) and the potential benefit of gaining various carbon and nitrogen sources needed for growth offers insights that support extending the resource allocation principle (which was originally established for cells grown on minimal media) to systems with complex nutritional environments.

### Limitation of protein-cost constraints

Compared with classic metabolic models, the key advance of resource allocation models is the inclusion of the costs of macromolecular expressions on top of the metabolism. Taking ME as an example, pathways with higher metabolic efficiency (high-yield) are usually ‘longer’ than less efficient (low-yield) pathways. Therefore, high-yield pathways are always coupled with higher protein costs. When the growth rate is maximised, this feature (i.e. high-yield-high-cost) governs the prediction of the metabolic switch from high-yield to low-yield pathways at increased growth rates. Although protein-cost constraints have been shown to efficiently improve the prediction of metabolic fluxes, this effectiveness can be easily spoiled if two alternative pathways hold similar protein costs. For example, pyruvate dehydrogenase (Pdh) and pyruvate formate lyase (Pfl) both convert pyruvate into acetyl-CoA with a cost of one enzyme. ME predicts the use of Pfl instead of Pdh, whilst the use of the latter was reported by fluxomics [54]. This outlier can be rectified by adding in regulatory rules, i.e. Pfl is activated only under anoxic conditions [82, 83]. However, the regulatory network is currently not in the scope of ME [84] and to our knowledge nor has it been systematically modelled in other resource allocation models. It would be valuable to construct a model that comprises both metabolic, expression machinery and regulatory networks, paving the way towards more reliable predictive modelling. Apart from adding regulations, such flux prediction issues could be amended by introducing a global view of the system (through e.g. adding appropriate constraints that describe the dynamics of the entire system) beyond the local behaviour of individual reactions.

### Implications for synthetic biology

Synthetic circuits compete with their host (and other circuits) for cellular resources required for their respective functions. This resource competition often leads to poor performance and unexpected behaviour of designed systems [85–87]. However, our growing understanding of microbial resource allocation can help us engineer systems with improved performance and predictability.

A first problem is that the introduction of synthetic circuits often results in growth defects because cellular resources are diverted away from biomass production. In the short term this can lead to loss of productivity; in the long term it can lead to complete loss of function due to evolution [88]. One approach to resolving this problem is to design synthetic circuits that use less cellular resources or avoid using the most limiting resources. The simplest way of doing this is by decreasing synthetic protein expression to relieve pressure on gene expression in general. This can be achieved either by swapping regulatory components or integrating constructs into the chromosome [89]. Alternatively, protein parts implementing memory or regulatory devices can be replaced with DNA or RNA parts, reducing load on translation which is usually the most limiting process in prokaryotic gene expression [90]. Many other functions, however, cannot be implemented without high enzyme concentrations. In such cases it is still possible to minimise the demand of synthetic circuits by using strong promoters and high copy number plasmids but with weak ribosome binding sites, again to limit the burden on translational machinery [91].

On the other hand, microbial hosts can be designed to increase their supply of cellular resources. In particular, it has been suggested that ‘lean-proteome’ strains with additional proteomic budgets can be generated by removing unnecessary but highly expressed genes [81]. According to the proteome allocation theory developed by Scott et al*.* [2], such strains should be capable of both improved growth and heterologous protein expression, and indeed significant increases in growth rate and biomass yield are observed upon deletion of non-essential genes in *Bacillus subtilis* [92, 93] and *E. coli* [94]. In a similar vein, a recent study takes advantage of the transcriptional regulatory network of *E. coli* to reduce unnecessary enzyme expression by deleting upstream transcriptional factors (rather than deleting the enzymes themselves). With only three genetic interventions, an optimized strain is generated with a demonstrably higher proteome budget and an increased production from a heterologous metabolic pathway [95]. One limitation of these approaches is that they only look at freeing up proteomic resources of completely unutilized genes, whereas significant proteomic resources may also be wasted on under-utilized genes [25].

A second problem is that resource competition couples the behaviour of circuits and hosts together in ways that can be difficult to predict. Synthetic circuits depend on host cellular resources such as ribosomes, amino acids and ATP to operate. The performance of synthetic circuits also depends on host physiological states, e.g. growth rate can affect the dilution of circuit components. However, formal models based on our growing biological understanding and tailored mathematical and computational methods are increasingly being used to accurately predict the outcome of circuit-host coupling [96]. For example, Weiße et al*.* develop a simple mechanistic model linking the expression of coarse-grained genes to microbial growth [97]. Their model not only recapitulates well established growth laws such as the linear relationship between ribosome levels and growth rate, but is also used to predict the amplitude and frequency of oscillations generated by a repressilator with varying transcriptional rates. Similarly, Liao et al*.* develop a multi-scale model of a fine-grained synthetic circuit operating inside a coarse-grained cell, and use their model to predict the bulk and single-cell dynamics of a bistable switch across nutritional and inductional parameters [98].

Alternatively, circuit-host coupling can be minimised by engineering microbial systems with orthogonal resource pools dedicated to synthetic functions [99]. For example, orthogonal transcriptional and degradation machinery have been implemented in *E. coli* using heterologous RNA polymerase and proteases taken from T7 bacteriophage and *Mesoplasma florum* bacterium respectively [100, 101]. However, the same strategy cannot be applied to make orthogonal translational machinery because the structure and function of ribosomes are highly conserved across species. Instead, orthogonal ribosomes have been implemented using synthetic 16S rRNA with high affinity for a non-canonical ribosome binding site. Despite the significant progresses in this area, it should be noted that these strategies achieve partial decoupling at best, as many important resource types such as amino acids and ATP are still shared with the cellular host and have no orthogonal counterpart [102].

## Conclusions

The study of cell growth and resource allocation contributes to the rising consensus on the key driver(s) of the phenotypic patterns of microorganisms. Cell growth and metabolic strategies are no longer considered to be dependent on the metabolic network only, but also subject to the cell’s active allocation of internal resources. Various resource allocation-based hypotheses have been developed to explain important biological phenomena, including (but not limited to) the pre-allocation of proteins and ribosomes, carbon catabolite repression, diauxie and co-utilisation of mixed carbon sources and the overflow metabolism. One important indication from these studies is that the biological importance of resource allocation can be diverse and may encompass maximising the growth rate, enabling rapid response to nutritional shifts and ensuring survival under harsh conditions. This thus supports the argument that the central role of resource allocation is to improve the overall cell’s fitness to the ever-changing environmental conditions. Resource allocation can facilitate different cellular objectives to provide the cell with different competitive advantages: pre-allocation offers the cell high adaptability to sudden nutritional shifts [25–28]; stress-driven proteome adjustments enable the cell strong vitality (survival capability) under harsh conditions [22]; and the utilisation of pathways with lower protein cost (e.g. the overflow metabolism) allows the cell to grow at fast rates [78]. Meanwhile, various mathematical models have been proposed to validate resource allocation associated hypotheses and experimental observations. We classified network-based resource allocation models into predictive models (including coarse-grained models and fine-grained models that are both generally FBA-based) and descriptive models that focus on elucidating the network topology through EFMA. Detailed overviews of each class of models can be found in [8–10]. Here, we complement previous work by emphasising the key feature of different resource allocation models and deciphering important modelling treatments (i.e. variable enzyme catalytic efficiencies in RBA and ME).

Despite these prominent achievements in this area, several important questions remain open. Firstly, among resource allocation-derived hypotheses for explaining the overflow metabolism, two competing explanations, i.e. the space limitation and machinery limitation, seem to be independently correct, making the physiological root cause of the overflow metabolism remain unclear. Secondly, the study in *lac* operon model system [77] raises the concern in the interpretation of the ‘protein cost’, which might not be limited to protein synthesis but also protein activation/function. Thirdly, previous research in resource allocation (both theoretical and experimental) mainly focuses on cell growth in minimal medium; the connection between cell growth and resource allocation in rich media remains an un-reclaimed field.

Finally, we would like to emphasise the importance of understanding resource allocation for synthetic biology, especially as we move towards implementing increasingly complex synthetic circuits. Widespread adoption of engineered microbes depends on our ability to design performant and predictable systems, which necessarily entails accounting for resource allocation. The realisation of this goal will likely involve a mixture of circuit, host and integrative design.

As the understanding of the mechanisms and utility of resource application in cellular systems further develops, we anticipate that mathematical modelling tools incorporating resource allocation will also further evolve to improve their mechanistic soundness. Together, these will facilitate the widening of the application of resource application mechanisms and principles in synthetic biology.

## Data Availability

Not applicable.

## Abbreviations

BMCs: bacterial microcompartments
cAMP: cyclic adenosine monophosphate
CCR: carbon catabolite repression
dFBA: dynamic flux balance analysis
EFM: elementary flux mode
EFMA: elementary flux mode analysis
FBA: flux balance analysis
ME: metabolic and macromolecular expression
NRPs: non-ribosomal proteins
Pdh: pyruvate dehydrogenase
Pfl: pyruvate formate lyase
RA: resource allocation
RBA: resource balance analysis
RNAPs: RNA polymerases
SASA: solvent accessible surface area
SNL: strictly nutrient-limited

## References

[1] Molenaar D, van Berlo R, de Ridder D, Teusink B (2009). Shifts in growth strategies reflect tradeoffs in cellular economics. Mol Syst Biol.

[2] Scott M, Gunderson CW, Mateescu EM, Zhang Z, Hwa T (2010). Interdependence of cell growth and gene expression: origins and consequences. Science.

[3] You C, Okano H, Hui S, Zhang Z, Kim M, Gunderson CW (2013). Coordination of bacterial proteome with metabolism by cyclic AMP signalling. Nature.

[4] Basan M, Hui S, Okano H, Zhang Z, Shen Y, Williamson JR (2015). Overflow metabolism in *Escherichia coli* results from efficient proteome allocation. Nature.

[5] Hui S, Silverman JM, Chen SS, Erickson DW, Basan M, Wang J (2015). Quantitative proteomic analysis reveals a simple strategy of global resource allocation in bacteria. Mol Syst Biol.

[6] Erickson DW, Schink SJ, Patsalo V, Williamson JR, Gerland U, Hwa T (2017). A global resource allocation strategy governs growth transition kinetics of Escherichia coli. Nature.

[7] Goelzer A, Fromion V (2017). Resource allocation in living organisms. Biochem Soc Trans.

[8] Yang L, Yurkovich JT, King ZA, Palsson BO (2018). Modeling the multi-scale mechanisms of macromolecular resource allocation. Curr Opin Microbiol.

[9] Basan M (2018). Resource allocation and metabolism: the search for governing principles. Curr Opin Microbiol.

[10] de Groot DH, Lischke J, Muolo R, Planqué R, Bruggeman FJ, Teusink B (2019). The common message of constraint-based optimization approaches: overflow metabolism is caused by two growth-limiting constraints. Cell Mol Life Sci.

[11] Pramanik J, Keasling JD (1998). Effect of Escherichia coli biomass composition on central metabolic fluxes predicted by a stoichiometric model. Biotechnol Bioeng.

[12] Taymaz-Nikerel H, Borujeni AE, Verheijen PJT, Heijnen JJ, van Gulik WM (2010). Genome-derived minimal metabolic models for *Escherichia coli* MG1655 with estimated in vivo respiratory ATP stoichiometry. Biotechnol Bioeng.

[13] Neidhardt FC, Ingraham JL, Schaechter M (1990). Physiology of the bacterial cell.

[14] Goelzer A, Fromion V (2011). Bacterial growth rate reflects a bottleneck in resource allocation. Biochim Biophys Acta Gen Subj.

[15] Dourado H, Lercher MJ (2020). An analytical theory of balanced cellular growth. Nat Commun.

[16] de Groot DH, Hulshof J, Teusink B, Bruggeman FJ, Planqué R (2020). Elementary growth modes provide a molecular description of cellular self-fabrication. PLoS Comput Biol.

[17] Gosset G (2005). Improvement of *Escherichia coli* production strains by modification of the phosphoenolpyruvate:sugar phosphotransferase system. Microb Cell Fact.

[18] Rosano GL, Ceccarelli EA (2014). Recombinant protein expression in *Escherichia coli*: advances and challenges. Front Microbiol.

[19] Shimizu K, Matsuoka Y (2019). Regulation of glycolytic flux and overflow metabolism depending on the source of energy generation for energy demand. Biotechnol Adv.

[20] Portnoy VA, Bezdan D, Zengler K (2011). Adaptive laboratory evolution—harnessing the power of biology for metabolic engineering. Curr Opin Biotechnol.

[21] Towbin BD, Korem Y, Bren A, Doron S, Sorek R, Alon U (2017). Optimality and sub-optimality in a bacterial growth law. Nat Commun.

[22] Radzikowski JL, Vedelaar S, Siegel D, Ortega ÁD, Schmidt A, Heinemann M (2016). Bacterial persistence is an active σS stress response to metabolic flux limitation. Mol Syst Biol.

[23] Yang JH, Bening SC, Collins JJ (2017). Antibiotic efficacy—context matters. Curr Opin Microbiol.

[24] Schmidt A, Kochanowski K, Vedelaar S, Ahrné E, Volkmer B, Callipo L (2016). The quantitative and condition-dependent *Escherichia coli* proteome. Nat Biotechnol.

[25] O’Brien EJ, Utrilla J, Palsson BO (2016). Quantification and classification of *E. coli* proteome utilization and unused protein costs across environments. PLoS Comput Biol.

[26] Li SH-J, Li Z, Park JO, King CG, Rabinowitz JD, Wingreen NS (2018). *Escherichia coli* translation strategies differ across carbon, nitrogen and phosphorus limitation conditions. Nat Microbiol.

[27] Mori M, Schink S, Erickson DW, Gerland U, Hwa T (2017). Quantifying the benefit of a proteome reserve in fluctuating environments. Nat Commun.

[28] Korem Kohanim Y, Levi D, Jona G, Towbin BD, Bren A, Alon U (2018). A bacterial growth law out of steady state. Cell Rep.

[29] New AM, Cerulus B, Govers SK, Perez-Samper G, Zhu B, Boogmans S (2014). Different levels of catabolite repression optimize growth in stable and variable environments. PLoS Biol.

[30] Bremer H, Dennis P (1996). *Escherichia coli* and Salmonella.

[31] Maaløe O (1979). Biological regulation and development.

[32] Venturelli OS, Tei M, Bauer S, Chan LJG, Petzold CJ, Arkin AP (2017). Programming mRNA decay to modulate synthetic circuit resource allocation. Nat Commun.

[33] Segall-Shapiro TH, Meyer AJ, Ellington AD, Sontag ED, Voigt CA (2014). A ‘resource allocator’ for transcription based on a highly fragmented T7 RNA polymerase. Mol Syst Biol.

[34] Zhou Y, Vazquez A, Wise A, Warita T, Warita K, Bar-Joseph Z (2013). Carbon catabolite repression correlates with the maintenance of near invariant molecular crowding in proliferating *E. coli* cells. BMC Syst Biol.

[35] Szenk M, Dill KA, de Graff AMR (2017). Why do fast-growing bacteria enter overflow metabolism? Testing the membrane real estate hypothesis. Cell Syst.

[36] Agapakis CM, Boyle PM, Silver PA (2012). Natural strategies for the spatial optimization of metabolism in synthetic biology. Nat Chem Biol.

[37] Wang X, Xia K, Yang X, Tang C (2019). Growth strategy of microbes on mixed carbon sources. Nat Commun.

[38] Görke B, Stülke J (2008). Carbon catabolite repression in bacteria: many ways to make the most out of nutrients. Nat Rev Microbiol.

[39] De Deken RH (1966). The crabtree effect: a regulatory system in yeast. Microbiology.

[40] Yu R, Nielsen J (2019). Big data in yeast systems biology. FEMS Yeast Res.

[41] Chen Y, Nielsen J (2019). Energy metabolism controls phenotypes by protein efficiency and allocation. Proc Natl Acad Sci.

[42] Vander Heiden MG, Cantley LC, Thompson CB (2009). Understanding the Warburg effect: the metabolic requirements of cell proliferation. Science.

[43] Shlomi T, Benyamini T, Gottlieb E, Sharan R, Ruppin E (2011). Genome-scale metabolic modeling elucidates the role of proliferative adaptation in causing the Warburg effect. PLoS Comput Biol.

[44] de Alteriis E, Cartenì F, Parascandola P, Serpa J, Mazzoleni S (2018). Revisiting the Crabtree/Warburg effect in a dynamic perspective: a fitness advantage against sugar-induced cell death. Cell Cycle.

[45] Vazquez A, Beg QK, Demenezes MA, Ernst J, Bar-Joseph Z, Barabasi AL (2008). Impact of the solvent capacity constraint on *E. coli* metabolism. BMC Syst Biol.

[46] Beg QK, Vazquez A, Ernst J, de Menezes MA, Bar-Joseph Z, Barabasi AL (2007). Intracellular crowding defines the mode and sequence of substrate uptake by *Escherichia coli* and constrains its metabolic activity. Proc Natl Acad Sci USA.

[47] Zhuang K, Vemuri GN, Mahadevan R (2011). Economics of membrane occupancy and respiro-fermentation. Mol Syst Biol.

[48] Vazquez A, Oltvai ZN (2016). Macromolecular crowding explains overflow metabolism in cells. Sci Rep.

[49] Woldringh CL, Binnerts JS, Mans A (1981). Variation in *Escherichia coli* buoyant density measured in Percoll gradients. J Bacteriol.

[50] Basan M, Zhu M, Dai X, Warren M, Sévin D, Wang Y-P (2015). Inflating bacterial cells by increased protein synthesis. Mol Syst Biol.

[51] Goelzer A, Fromion V, Scorletti G (2011). Cell design in bacteria as a convex optimization problem. Automatica.

[52] Goelzer A, Muntel J, Chubukov V, Jules M, Prestel E, Nölker R (2015). Quantitative prediction of genome-wide resource allocation in bacteria. Metab Eng.

[53] Thiele I, Fleming RMT, Que R, Bordbar A, Diep D, Palsson BO (2012). Multiscale modeling of metabolism and macromolecular synthesis in *E. coli* and its application to the evolution of codon usage. PLoS ONE.

[54] O’Brien EJ, Lerman JA, Chang RL, Hyduke DR, Palsson BO (2013). Genome-scale models of metabolism and gene expression extend and refine growth phenotype prediction. Mol Syst Biol.

[55] Maitra A, Dill KA (2015). Bacterial growth laws reflect the evolutionary importance of energy efficiency. Proc Natl Acad Sci.

[56] Chen T, He HL, Church GM. Modeling gene expression with differential equations. In: Proceedings of pacific symposium on biocomputing (PSB’99). Singapore: World Scientific; 1998. p. 29–40. 10.1142/9789814447300_0004. 10380183

[57] Tchourine K, Poultney CS, Wang L, Silva GM, Manohar S, Mueller CL (2014). One third of dynamic protein expression profiles can be predicted by a simple rate equation. Mol BioSyst.

[58] Maurizi MR (1992). Proteases and protein degradation in *Escherichia coli*. Experientia.

[59] Dressaire C, Gitton C, Loubière P, Monnet V, Queinnec I, Cocaign-Bousquet M (2009). Transcriptome and proteome exploration to model translation efficiency and protein stability in *Lactococcus lactis*. PLOS Comput Biol.

[60] Nóbel F, Picó J (2020). Resources allocation explains the differential roles of RBS and promoter strengths in cell mass distribution and optimal protein expression productivity. bioRxiv.

[61] Orth JD, Thiele I, Palsson BØ (2010). What is flux balance analysis?. Nat Biotechnol.

[62] Mori M, Hwa T, Martin OC, De Martino A, Marinari E (2016). Constrained allocation flux balance analysis. PLoS Comput Biol.

[63] Zeng H, Yang A (2019). Modelling overflow metabolism in *Escherichia coli* with flux balance analysis incorporating differential proteomic efficiencies of energy pathways. BMC Syst Biol.

[64] Zeng H, Yang A (2019). Quantification of proteomic and metabolic burdens predicts growth retardation and overflow metabolism in recombinant *Escherichia coli*. Biotechnol Bioeng.

[65] Schomburg I, Chang A, Placzek S, Söhngen C, Rother M, Lang M (2012). BRENDA in 2013: integrated reactions, kinetic data, enzyme function data, improved disease classification—new options and contents in BRENDA. Nucleic Acids Res.

[66] Sánchez BJ, Zhang C, Nilsson A, Lahtvee P-J, Kerkhoven EJ, Nielsen J (2017). Improving the phenotype predictions of a yeast genome-scale metabolic model by incorporating enzymatic constraints. Mol Syst Biol.

[67] Karr JR, Sanghvi JC, Macklin DN, Gutschow MV, Jacobs JM, Bolival B (2012). A whole-cell computational model predicts phenotype from genotype. Cell.

[68] Yang L, Tan J, O’Brien EJ, Monk JM, Kim D, Li HJ (2015). Systems biology definition of the core proteome of metabolism and expression is consistent with high-throughput data. Proc Natl Acad Sci.

[69] Peyraud R, Cottret L, Marmiesse L, Gouzy J, Genin S (2016). A resource allocation trade-off between virulence and proliferation drives metabolic versatility in the plant pathogen *Ralstonia solanacearum*. PLoS Pathog.

[70] Waldherr S, Oyarzún DA, Bockmayr A (2015). Dynamic optimization of metabolic networks coupled with gene expression. J Theor Biol.

[71] Mahadevan R, Edwards JS, Doyle FJ (2002). Dynamic flux balance analysis of diauxic growth in *Escherichia coli*. Biophys J.

[72] Schuster S, Fell DA, Dandekar T (2000). A general definition of metabolic pathways useful for systematic organization and analysis of complex metabolic networks. Nat Biotechnol.

[73] Carlson RP (2007). Metabolic systems cost-benefit analysis for interpreting network structure and regulation. Bioinformatics.

[74] Wortel MT, Noor E, Ferris M, Bruggeman FJ, Liebermeister W (2018). Metabolic enzyme cost explains variable trade-offs between microbial growth rate and yield. PLoS Comput Biol.

[75] de Groot DH, van Boxtel C, Planqué R, Bruggeman FJ, Teusink B (2019). The number of active metabolic pathways is bounded by the number of cellular constraints at maximal metabolic rates. PLoS Comput Biol.

[76] Lerman JA, Hyduke DR, Latif H, Portnoy VA, Lewis NE, Orth JD (2012). In silico method for modelling metabolism and gene product expression at genome scale. Nat Commun.

[77] Eames M, Kortemme T (2012). Cost-benefit tradeoffs in engineered lac operons. Science (80- ).

[78] Mori M, Marinari E, De Martino A (2019). A yield-cost tradeoff governs *Escherichia coli*’s decision between fermentation and respiration in carbon-limited growth. npj Syst Biol Appl.

[79] Cheng C, O’Brien EJ, McCloskey D, Utrilla J, Olson C, LaCroix RA (2019). Laboratory evolution reveals a two-dimensional rate-yield tradeoff in microbial metabolism. PLoS Comput Biol.

[80] Reimers A-M, Knoop H, Bockmayr A, Steuer R (2017). Cellular trade-offs and optimal resource allocation during cyanobacterial diurnal growth. Proc Natl Acad Sci.

[81] Zampieri M, Hörl M, Hotz F, Müller NF, Sauer U (2019). Regulatory mechanisms underlying coordination of amino acid and glucose catabolism in *Escherichia coli*. Nat Commun.

[82] Orth JD, Palsson BØ, Fleming RMT (2010). Reconstruction and use of microbial metabolic networks: the core *Escherichia coli* metabolic model as an educational guide. EcoSal Plus.

[83] Sawers G, Watson G (1998). A glycyl radical solution: oxygen-dependent interconversion of pyruvate formate-lyase. Mol Microbiol.

[84] King ZA, O’Brien EJ, Feist AM, Palsson BO (2017). Literature mining supports a next-generation modeling approach to predict cellular byproduct secretion. Metab Eng.

[85] Borkowski O, Ceroni F, Stan G-B, Ellis T (2016). Overloaded and stressed: whole-cell considerations for bacterial synthetic biology. Curr Opin Microbiol.

[86] Boo A, Ellis T, Stan G-B (2019). Host-aware synthetic biology. Curr Opin Syst Biol.

[87] Cardinale S, Arkin AP (2012). Contextualizing context for synthetic biology: identifying causes of failure of synthetic biological systems. Biotechnol J.

[88] Nikolados E-M, Weiße AY, Ceroni F, Oyarzún DA (2019). Growth defects and loss-of-function in synthetic gene circuits. ACS Synth Biol.

[89] Santos CNS, Regitsky DD, Yoshikuni Y (2013). Implementation of stable and complex biological systems through recombinase-assisted genome engineering. Nat Commun.

[90] Brophy JAN, Voigt CA (2014). Principles of genetic circuit design. Nat Methods.

[91] Ceroni F, Algar R, Stan G-B, Ellis T (2015). Quantifying cellular capacity identifies gene expression designs with reduced burden. Nat Methods.

[92] Fischer E, Sauer U (2005). Large-scale in vivo flux analysis shows rigidity and suboptimal performance of *Bacillus subtilis* metabolism. Nat Genet.

[93] Muntel J, Fromion V, Goelzer A, Maaβ S, Mäder U, Büttner K (2014). Comprehensive absolute quantification of the cytosolic proteome of bacillus subtilis by data independent, parallel fragmentation in liquid chromatography/mass spectrometry (LC/MSE). Mol Cell Proteom.

[94] D’Souza G, Waschina S, Pande S, Bohl K, Kaleta C, Kost C (2014). Less is more: selective advantages can explain the prevalent loss of biosynthetic genes in bacteria. Evolution (N Y).

[95] Lastiri-Pancardo G, Mercado-Hernández JS, Kim J, Jiménez JI, Utrilla J (2020). A quantitative method for proteome reallocation using minimal regulatory interventions. Nat Chem Biol.

[96] Nikolados E-M, Weiße AY, Oyarzún DA. Prediction of cellular burden with host-circuit models. arXiv e-prints. 2020. arXiv:2004.00995. 10.1007/978-1-0716-1032-9_13 33405227

[97] Weiße AY, Oyarzún DA, Danos V, Swain PS. Mechanistic links between cellular trade-offs, gene expression, and growth. Proc Natl Acad Sci. 2015;112:E1038 LP-E1047. doi:10.1073/pnas.1416533112. 10.1073/pnas.1416533112 PMC4352769 25695966

[98] Liao C, Blanchard AE, Lu T (2017). An integrative circuit–host modelling framework for predicting synthetic gene network behaviours. Nat Microbiol.

[99] Liu CC, Jewett MC, Chin JW, Voigt CA (2018). Toward an orthogonal central dogma. Nat Chem Biol.

[100] Meyer AJ, Ellefson JW, Ellington AD (2015). Directed evolution of a panel of orthogonal T7 RNA polymerase variants for in vivo or in vitro synthetic circuitry. ACS Synth Biol.

[101] Cameron DE, Collins JJ (2014). Tunable protein degradation in bacteria. Nat Biotechnol.

[102] Darlington APS, Kim J, Jiménez JI, Bates DG (2018). Dynamic allocation of orthogonal ribosomes facilitates uncoupling of co-expressed genes. Nat Commun.

